# The Effect of Maternal Stress on 11beta-Hydroxysteroid Dehydrogenase Activity During Pregnancy: Evidence for Potential Pregnancy Complications and Consequences on Fetal Development and Metabolism

**DOI:** 10.3390/ijms262211071

**Published:** 2025-11-16

**Authors:** Polina Pavli, George Mastorakos, Makarios Eleftheriades, Georgios Valsamakis

**Affiliations:** 1 2nd Department of Obstetrics and Gynecology, National and Kapodistrian University of Athens, “Aretaieion” University Hospital, 11528 Athens, Greece; polinapav@med.uoa.gr (P.P.); makarios@hotmail.co.uk (M.E.); 2 Unit of Endocrinology, Diabetes Mellitus and Metabolism, Aretaieion Hospital, Athens Medical School, National and Kapodistrian University of Athens, 11528 Athens, Greece; mastorakg@gmail.com

**Keywords:** maternal stress, 11β-hydroxysteroid dehydrogenase enzyme activity, preeclampsia, preterm birth, intrauterine growth restriction, small for gestational age

## Abstract

Τhe intrauterine environment has a strong connection with the growing fetus and possible effects that can continue up to adulthood. Currently, stress is conceptualized as a modern teratogen. The overwhelming majority of studies indicate that maternal stress during pregnancy may have effects on pregnancy outcomes and fetal development, with long-lasting consequences on child and adult vulnerability to disease. Glucocorticoids are essential for regulating fetal development, growth, and metabolism. The two isoforms of 11beta-hydroxysteroid dehydrogenase enzyme (11β-HSD) mediate and regulate glucocorticoid actions and biological activity. It has not yet been fully elucidated whether maternal stress during pregnancy affects 11β-HSD isoenzyme activity and expression and results in possible adverse effects on fetal development, metabolism, and pregnancy outcomes. This review examines a possible pathophysiological mechanism by which maternal stress during pregnancy affects placental 11β-HSD isoenzyme activity, thereby causing adverse effects on the physiological status of pregnancy, fetal development, and metabolism. Furthermore, the main outcome of the review is the following: chronic and acute maternal stress during pregnancy affects the activity and the expression of placental 11β-HSD isoenzymes and has possible subsequent unfavorable results on preeclampsia, preterm birth, and fetuses with intrauterine growth restriction (IUGR) or small for gestational age (SGA) fetuses.

## 1. Introduction

Τhe intrauterine environment is of utmost importance, as it is closely linked to the developing fetus and one’s general health throughout life [1]. Emerging evidence indicates that exposure to a detrimental intrauterine environment manifests an increased risk of cardiometabolic disorders such as high blood pressure, ischemic heart disease, hyperlipidemia, impaired insulin response, type 2 diabetes mellitus, and excess body weight, as well as reduced birth weight and hormonal abnormalities leading to disease till adulthood [2,3,4,5]. Recently, human studies also suggested that the intrauterine environment may also affect the likelihood of developing polycystic ovary syndrome and hyperandrogenism in later life [6,7].

Currently, stress caused by social or psychological factors and anxiety during pregnancy constitute a worldwide, expanding concern, while stress is increasingly recognized as a modern teratogen [8,9]. A substantial body of literature demonstrates that maternal stress during pregnancy can profoundly affect fetal development and have several consequences on child and adult vulnerability to disease regarding emotional, cognitive, and behavioral outcomes and metabolic functioning [10,11,12]. Other related outcomes may be the increased incidence of pregnancy complications [13], effects on anthropometric measurements of the newborn, and effects regarding infant cortisol response and stress reactivity [14,15]. It is well established that stress stimulates gluconeogenesis through the stimulation of the hypothalamic-pituitary-adrenal (HPA) axis. The production of glucocorticoid hormones and corticotrophin-releasing hormone (CRH) mediates stress-induced hyperglycemia, while there is also the issue of low-grade chronic inflammation; this whole process eventually causes more damage through intrauterine life [16].

Glucocorticoids play a major role in the developmental programming οf the fetus. Glucocorticoid actions are mediated and regulated by the two isoforms of 11beta-hydroxysteroid dehydrogenase (11β-HSD) [1]. 11β-HSD type 1 functions mainly as a reductase by reactivating glucocorticoids from their inactive 11-keto metabolites [17], while 11β-HSD type 2 acts as an oxidase by deactivating cortisol to cortisone [18]. During pregnancy, the maternal HPA axis experiences significant modifications, causing hypercortisolemia [1]. It has already been established that 11β-HSD isoenzymes are implicated in the pathogenesis of several diseases and complications of pregnancy [1]. Moreover, increased concentrations of glucocorticoids in utero may be damaging to placental development and fetal growth [19,20].

However, the exact underlying pathophysiological mechanisms through which maternal stress during pregnancy affects fetal development, metabolism, and subsequent health outcomes are not yet fully elucidated [21]. Most of the data that supports the glucocorticoid hypothesis is primarily derived from animal studies [22,23]. Moreover, 11β-HSD isoenzymes are not only produced through the HPA axis but also present in the placenta and other tissues [17,18]. This review will mainly investigate the effects of maternal stress during pregnancy on the placental isoenzymes. It remains unclear whether maternal stress during pregnancy affects placental 11β-HSD isoenzymes activity or expression, causing adverse effects on the physiological status of pregnancy, fetal development, and metabolism.

In this review, we will investigate the emerging evidence on the effects of maternal stress (chronic and acute) during pregnancy on the activity of placental 11β-HSD isoenzymes and cortisol metabolism and their possible subsequent unfavorable results on pregnancy outcomes, fetal development, and metabolism.

## 2. Materials and Methods

To identify publications regarding the effects of maternal stress (chronic and acute) during pregnancy upon the activity of 11β-HSD isoenzymes and their possible subsequent unfavorable results, a systematic literature search for human and animal studies was conducted in three electronic databases (PubMed, Cochrane, and Medline) until October 2025. The key terms used were “maternal stress” AND “11β-HSD activity”, AND “pregnancy outcomes” OR “fetal development” OR “fetal metabolism”. We found a total of 146 articles, including all types of studies. The papers that were finally included in our review comprised every type of article related to maternal stress during pregnancy.

## 3. Physiology of Stress and Cortisol Metabolism

The intrauterine environment plays a crucial role in fetal growth and significantly influences an infant’s later health status [1]; it is also linked to an increased possibility of suffering from metabolic and other diseases during life.

Stress is defined as the process of adjustment of the human organism, through which it reacts to a difficult situation in order to face challenges and threats throughout life [24]. It is separated into acute and chronic stress. Acute stress is defined as the human body’s transient exposure either to a variety of stressors that act simultaneously or to a natural event of short-term action [25]. Chronic stress is the human body’s physiological or psychological reaction to a long-term internal or external stress stimulus [25]. Acute and chronic stress might be estimated based on the STAI state-trait (Spielberger State-Trait Anxiety Inventory) questionnaires, which evaluate both types of stress based on 20 questions for each one [26]. These questionnaires are considered reliable for the assessment of acute and chronic stress during pregnancy. The physiological reaction of the human body in stressful situations is called the “fight or flight” response and occurs after the release of corticosteroid hormones [25]. As the primary stress hormone, cortisol has multiple effects on human physiology: the development of insulin resistance, hyperglycemia, abdominal obesity, an increase in blood pressure, loss of muscle mass, osteoporosis, skin striae, reduced connective tissue production, poor wound healing, suppression of the immune system, and an increase in infections [27]. Specifically, during pregnancy, the activity of cortisol is associated with fetal development and anthropometric characteristics of birth, such as birth weight, birth length, adequate development of fetal lungs, effects on the neurons, and normal onset of labor [28]. Excessive corticoid concentrations are associated with abnormalities in fetal development [29].

The metabolism of cortisol occurs through multiple enzymatic pathways. The major enzymes involved in the metabolism of cortisol to inert cortisone and vice versa are 11β-HSD type 1 and 11β-HSD type 2 [17,18]. Specifically, 11β-HSD type 1 reduces cortisone to active cortisol, and its concentration is increased in metabolic tissues, such as the liver, the central nervous system, and fat tissue. 11β-HSD type 2 reduces cortisol to inactive cortisone, and its concentration is increased in tissues, which are simultaneously “target tissues” for aldosterone, such as the liver, kidneys, lungs, colon, salivary glands, central nervous system, and the placenta [17,18,30].

The activity of the enzymes 11β-HSD type 1 and 11β-HSD type 2 is related to the development of several diseases throughout life: insulin resistance, obesity or metabolic syndrome, polycystic ovarian syndrome, osteoporosis, breast cancer, glaucoma, hypertension, cognitive and neurobehavioral disorders, and intrauterine growth restriction [1]. The enzyme 11β-HSD type 1 can be found on the maternal side of the placenta, mainly in stromal cells but also in epithelial cells, embryonic vessels of the chorionic villi, extravillous trophoblast, and amniotic epithelial cells [20,30]. Moreover, the enzyme 11β-HSD type 2 can be found at increased concentrations in the stromal cells of the placenta, in the syncytiotrophoblast of the chorionic villi, and also in the placental epithelium [20,30].

## 4. Maternal Stress During Pregnancy and Consequences on the Mother and Fetus

### 4.1. Maternal Stress During Pregnancy and Pregnancy Complications

#### 4.1.1. Maternal Cardiovascular Diseases—Hypertension and Preeclampsia

Chronic stress during pregnancy may lead to several endocrine alterations in the mother, such as changes in the secretion of adrenocorticotropic hormone (ACTH), glucocorticoids, and catecholamines [31]. Chronic stress is linked with changes in cortisol and epinephrine concentrations; the latter could lead to poor pregnancy outcomes and cardiovascular diseases during pregnancy [32].

As far as acute stress is concerned, stressful job characteristics have shown connections with pregnancy-induced hypertension. Psychosocial job stressors might lead to an increased risk of pregnancy-induced hypertension among pregnant women who still work. In particular, hypertension during pregnancy was shown to be associated with lower-status jobs and higher-status jobs, but in the latter case, gestational hypertension was associated with job stressors [33]. Depression and anxiety during early pregnancy may modify the release of hormones and neuroendocrine transmitters, potentially heightening the risk for pregnancy-related hypertension [34].

Pregnant women who eventually develop preeclampsia often show increased placental CRH concentrations at 18–20 weeks of gestation [34]. Depression and anxiety during the beginning of pregnancy seem to be connected to a greater likelihood of preeclampsia occurring during a later phase of pregnancy [34]. Another previous study of 4314 women with singleton pregnancies presented the following critical finding: chronic hypertension, combined with high chronic stress levels, before or during pregnancy, is significantly linked with an increased risk of preeclampsia [35]. A more recent study suggested that chronic and acute stress during pregnancy may be linked to gestational hypertension and preeclampsia [36].

#### 4.1.2. Preterm Birth

Chronic maternal stress during pregnancy might be associated with stress-related modifications of plasma concentrations of catecholamines [37]. Maternal stress during pregnancy and CRH concentrations, especially during the second trimester, may be used as markers for increased risk of preterm birth [37]. A study of 340 women examined maternal stress during pregnancy as a risk factor for preterm birth. Participants had undergone stress during pregnancy, with depression, anxiety, or other psychiatric diagnoses, or self-reported stress due to the pregnancy itself [38]. The results stated that preterm delivery was more commonly mentioned among women who experienced stress during pregnancy. In detail, 54% of the women who experienced stress during pregnancy also experienced preterm delivery. Thus, stress was shown to contribute significantly to the risk of preterm delivery [38]. Morgan et.al. found that chronic and acute stress during pregnancy may be linked to preterm birth [36].

#### 4.1.3. Spontaneous Abortion

Chronic stress and recent stressful events during pregnancy are both linked with an increased risk of spontaneous abortion [39]. Maternal stress is thought to be able to cause changes in the placenta that could affect fetal neurodevelopment and also lead to high cortisol concentrations by decreasing the activity of enzyme 11β-HSD type 2. This fact supports epidemiological data showing that 12% of all clinically recognized pregnancies resulted in spontaneous abortion [40].

#### 4.1.4. Maternal Metabolism—Gestational Diabetes Mellitus

Chronic stress maintains a generalized catabolic condition. Thus, it leads to loss of lean body mass, elevated central fat deposition, and decreased insulin sensitivity [31]. There are studies suggesting that anxiety and depression during pregnancy might result in induced sympathetic adrenal medulla system, promoted secretion of ACTH, glucocorticoid, glucagon, and catecholamines, and accelerated gluconeogenesis [41].

Chronic maternal stress during pregnancy is linked with decreased insulin sensitivity of the mother [42], and it was also identified as a potential risk factor for gestational diabetes mellitus (GDM) [16]. Researchers have investigated anxiety and depression during pregnancy as a predisposing factor for the prevalence of GDM [43,44,45].

In a randomized controlled study, the researchers proposed that pregnant women with negative emotions present increased plasma glucose concentrations, with more difficult glycemic control [46]. Additionally, HbA1c was positively correlated with high anxiety and depression scores in pregnant women with GDM [43], while some studies that examined pregnant women with GDM, based on the trait anxiety subscale in STAI, suggest that HbA1c levels were higher in the high trait anxiety group when compared to the low trait anxiety group [47].

Eventually, pregnant women diagnosed with GDM with high anxiety and depression status had significantly higher adverse maternal and infant outcomes, such as significantly lower neonatal birth weight or macrosomia, premature births, postpartum hemorrhage or infection, neonatal asphyxia, and neonatal hypoglycemia [48,49].

### 4.2. Maternal Stress During Pregnancy and Fetal Development

#### 4.2.1. In Utero

A suboptimal in utero environment resulting from increased maternal stress during pregnancy may have several impacts on the pregnancy and future unfavorable outcomes of the offspring [50]. Firstly, excessive concentrations of glucocorticoids might lead to intrauterine growth restriction (IUGR) [51]. Newborns with a birth weight and/or birth length below the third percentile for their gestational age are classified as presenting with intrauterine growth restriction.

#### 4.2.2. At Birth

During pregnancy, anxiety and depression of the mother contribute to diminished birth weight and reduced head circumference at birth [52,53]. The possibility of delivering a baby with a low birth weight is higher when maternal stress occurs during the first quarter of pregnancy [54].

Chronic maternal stress during pregnancy compromises the regulation of normal hormonal activity; excess of CRH and other hormones, such as cortisol and metenkephalin, may decrease birth weight and growth rate among stress-exposed infants [54,55]. Borders et al. investigated low-income women and showed that a stressful environment may be linked with low birth weight at birth [56]. Zhu et al., in a cohort involving 1800 women, proposed that for each unit increase in maternal stress during the first trimester, infant birth weight would be decreased by approximately 99 g [57]. In another prospective longitudinal study, mothers in late pregnancy and their singletons were investigated regarding the impact of chronic maternal stress upon gestational age and anthropometric measurements at delivery [58]. The results showed that chronic maternal stress during late gestation was associated with a marked decline in birth weight, length, and head circumference [58].

However, an association between stressful events during pregnancy and fetal growth has not been found by all researchers. Another multicenter study showed no evidence that either perceived stress or depressive symptoms during pregnancy result in altered fetal weight or anthropometric parameters [59]. Their findings indicate that higher levels of depressive symptoms and stress, as quantified by the Edinburgh Postpartum Depression Survey (EPDS) and the Cohen Perceived Stress Scale (PSS), respectively, are not associated with altered fetal growth throughout gestation, as this is defined by fetal weight, biparietal diameter, head circumference, abdominal circumference, and femur length [59]. Additionally, more research examining stressful events and perceived maternal stress has not identified any connection between maternal stress and fetal growth restriction [60,61].

#### 4.2.3. Childhood Pathologies

Increased fetal glucocorticoids caused by maternal stress and depression may affect the development of the fetal hypothalamus and pituitary, potentially reprogramming the HPA axis and also possibly leading to behavioral changes that impact the offspring’s neurogenesis [54,62].

Chronic maternal stress and excess amounts of CRH and cortisol may alter personality and also predispose infants to health and mental problems, such as attention deficit, hyperactivity, anxiety, and depression [63,64]. Increased stress during pregnancy results in affected neurodevelopment and impaired behavior in stressful conditions [63], while it can also be an important risk factor for schizophrenia and depression [65]. Clinical neurological evaluations present with lower scores in infants who experienced increased prenatal stress [55].

Additionally, the children of mothers who experienced severe acute stress during pregnancy are at higher risk of presenting with high body mass index (BMI) later in life and becoming overweight [66]. Thus, maternal stress during pregnancy may induce the development of metabolic syndrome. The increased exposure of the mother to psychosocial stress during pregnancy may also lead to decreased insulin sensitivity of the offspring [11].

### 4.3. Maternal Stress During Pregnancy and Changes in Fetal Metabolism

In cases of increased maternal stress during pregnancy, fetuses are not protected by the increased concentrations of cortisol [67]. In one study, it was suggested that umbilical cord plasma CRH concentrations were markedly higher in growth-retarded fetuses when compared to normal fetuses, accompanied by a tendency of increased umbilical cord cortisol concentrations [68]. Regarding other published data, increased maternal chronic stress, as defined by the STAI trait score, seems to be associated with increased fetal cortisol, glucose, and c-peptide concentrations. In detail, increased maternal STAI trait scores, especially during the second trimester of pregnancy, are linked with umbilical cord glucose and c-peptide concentrations. Regarding the third trimester, maternal STAI trait scores are also associated with umbilical cord c-peptide, glucose, insulin, and cortisol concentrations [69].

Regarding acute maternal stress during pregnancy, there is published data based on observations of mothers and their babies, who developed post-traumatic stress disorder (PTSD) due to September 11, compared to mothers, and their babies, who did not develop PTSD. Babies born to mothers with PTSD, particularly those exposed to stress during the third trimester, exhibited significantly lower cortisol concentrations. Acute maternal stress experienced during different trimesters may alter the HPA axis responsiveness in the offspring [70].

## 5. Maternal Stress During Pregnancy and Changes in the Expression and/or Activity of 11β-HSD Isoenzymes

An unfavorable intrauterine environment may affect the expression and activity of 11β-HSDs in multiple ways. According to a recent review, 11β-HSD type 2 may be used as a molecular target for fetal toxicity that affects its development, while 11β-HSD type 1 may be used as a therapeutic target to prevent several diseases [71].

A previous rat model examined maternal exposure to restrained stress during pregnancy regarding developmental and metabolic abnormalities of the offspring. In detail, the researchers examined the effects of maternal stress during pregnancy on hepatic 11β-HSD type 1 levels and also on the liver function of the offspring. In particular, researchers concluded that maternal restrained stress during pregnancy in mice is linked with increased levels of 11β-HSD type 1, and through this pathway, it may induce metabolic syndromes, such as increased lipid accumulation in the livers of the offspring of those mice [72].

Chronic maternal stress during pregnancy may deplete the protective nature of 11β-HSD type 2, leading to increased infant vulnerability and high concentrations of maternal cortisol. Moreover, previous studies show that maternal stress during pregnancy might lead to a down-regulation of 11β-HSD type 2 enzyme. Chronic maternal stress was investigated in an animal study with Long Evans rats regarding 11β-HSD type 2 mRNA in the placenta and fetal brain and the possible epigenetic pathways. Overall, the findings imply that chronic prenatal maternal stress indeed alters 11β-HSD type 2 gene expression through DNA methylation [73]. Larger studies are required to validate these findings, while another study recently presented intriguing results regarding the outcomes of maternal distress on 11β-HSD type 2 methylation [74]. Another study found that chronic maternal stress during pregnancy was negatively correlated with placental 11β-HSD type 2 mRNA expression, with no significant differences between male and female fetuses. Results were also significant for state anxiety, defined as acute maternal stress during pregnancy [67]. A recent meta-analysis involving a total of 1869 participants suggested that prenatal psychological distress (PPD) disorders are linked weakly with the placental 11β-HSD type 2 gene expression; in detail, lower placental 11β-HSD type 2 concentrations were detected in cases where PPD exposure happened during the third trimester [75]. The Mercy Pregnancy and Emotional Wellbeing Study investigated placental 11β-HSD type 2 expression among 33 pregnant women, who were followed up at 12–18 and 28–34 weeks of gestation. The findings presented negative correlations between 11β-HSD type 2 expression and both the STAI state-trait anxiety inventory and EPDS, in the first and third trimesters of pregnancy, with the associations being particularly prominent during late gestation [76].

A prior animal study examined the consequences of acute and chronic stress during the third week of pregnancy on placental 11β-HSD type 2 activity in rats. The results indicated that acute stress on gestational day 20 caused an immediate, significant increase in the placental 11β-HSD type 2 activity. In contrast, chronic stress from day 14 to day 19 of gestation did not significantly alter the basal 11β-HSD type 2 activity. However, chronic stress reduced the ability to up-regulate the placental 11β-HSD type 2 activity by almost 90% [77]. Data from recent research suggest that the ability to up-regulate 11β-HSD type 2 expression in response to acute stress diminishes as long as maternal stress during pregnancy becomes more chronic [74]. In addition, a recent rat model suggested that increased glucocorticoid concentrations of the mother due to stress could regulate, in a sex-specific manner, placental 11β-HSD type 2 expression. In detail, the researchers induced maternal glucocorticoid levels through prenatal caffeine exposure, using caffeine as a common stressor. The results stated that the expression of 11β-HSD type 2 was diminished and negatively correlated with the maternal and fetal glucocorticoid levels. However, this suggestion was only related to the male placentas that were exposed to the prenatal caffeine dosage [78]. Another recent animal model used preconception cold exposure as the common stressor of the research. The researchers exposed thirty rats to cold during preconception, or at three weeks of pregnancy, for a week in total, and compared them to controls. Their conclusions suggested that preconception cold exposure, used as a stressor, resulted in increased glucocorticoid levels throughout pregnancy and was linked to increased diastolic blood pressure in the rat cold exposure group when compared to control rats. When cold exposure was used as a stressor during the second week of pregnancy, this was associated with significantly lower placental weight. This could mean that stress during early pregnancy may have negative effects on placental vascularization and structure. Additionally, cold exposure as a stressor during the third week of pregnancy was associated with significantly lower fetal weight, and lastly, preconception cold exposure as a prenatal stressor was associated with significantly decreased placental levels of 11β-HSD type 2 [79].

## 6. 11β-HSD Type 1 and Type 2 Dysfunction Leading to Pregnancy Complications and Fetal Developmental and Metabolic Abnormalities

### 6.1. 11β-HSD Type 1 and Type 2 Dysfunction and Pregnancy Complications

A prior study indicated that polymorphisms within the 11β-HSD type 1 gene are linked with an increased risk of pregnancy-induced hypertension and preeclampsia [80]. Concentrations of 11β-HSD type 1 were higher in the placenta of pregnancies with preeclampsia when compared to controls. The results of a prior study highlight the predominance of 11β-HSD type 1 in early decidual cells [81]. Furthermore, ovarian hormones and inflammatory cytokines seem to up-regulate 11β-HSD type 1, while an elevated expression of 11β-HSD type 1 has also been reported in pregnancies with preeclampsia [81]. More recently, the inhibition of the 11β-HSD type 1 enzyme seemed to provide a potential therapeutic target for preterm birth [82].

11β-HSD type 2 expression and activity seem to be decreased in preeclampsia. Pro-inflammatory cytokines elevated in preeclampsia are among several signals that may modulate the 11β-HSD type 2 activity [83]. Furthermore, 11β-HSD type 2 activity is lower in preeclampsia, while at the same time, cortisol concentrations in the umbilical cord seem elevated, thus also suggesting the affected placental activity of the isoenzyme [84,85,86,87]. In contrast, other studies mention the increased activity of 11β-HSD type 2 enzyme in preeclampsia regardless of the possible small for gestational age (SGA) condition [88]. Lastly, another study of 45 pregnant women observed an important difference in GDM: an attributable down-regulation of 11b-HSD type 1 and up-regulation of other isoenzymes [89].

### 6.2. 11β-HSD Type 1 and Type 2 Dysfunction and Fetal Development

Reduced 11β-HSD type 1 activity might be found in placentas from human pregnancies with SGA fetuses [90]. 11β-HSD type 2 activity is shown to be suppressed in pregnancy-related abnormal fetal growth. Compared to normal pregnancies, placental 11β-HSD type 2 activity seems to be reduced in IUGR pregnancies in late gestation [91]. Published data have also highlighted a connection between 11β-HSD type 2 activity and fetal weight. Animal and human studies have shown that 11β-HSD type 2 deficiency during pregnancy results in low birth weight [92,93,94]. Moreover, recent studies have shown that mice 11β-HSD2 (−/−) present with low birth weight and increased anxiety [95].

### 6.3. 11β-HSD Type 1 and Type 2 Function and Changes in Fetal Metabolism

Tissue-specific dysregulation of 11β-HSD type 1 might be linked with metabolic disorders in the offspring of mothers suffering from GDM. In previous animal studies, offspring of diabetic mothers have presented with insulin resistance and glucose intolerance, as well as significantly increased 11β-HSD type 1 mRNA and activity in adipose tissue and liver. Therefore, 11β-HSD type 1 activity and expression in these tissues might play a role in the possible onset of metabolic syndrome in the offspring of diabetic mothers [96]. Regarding 11β-HSD type 2, there is a recent study that investigated placental 11β-HSD2 expression after collecting human placenta and umbilical cord blood. The samples were from women who had no prenatal use of synthetic glucocorticoids. The results showed that the placental expression of 11β-HSD type 2 was significantly correlated with umbilical cord cortisol concentrations and birth weight only in male newborns. No such correlation was shown regarding the female ones [78].

## 7. Discussion

In this review, after a literature search of studies that included humans or animals, we selectively identified all evidence that indicates a pathophysiological mechanism of stress upon possible subsequent unfavorable results of pregnancy and the fetus through changes in placental 11β-HSD isoenzyme activity and expression. The possible subsequent outcomes may concern maternal cardiovascular diseases, such as hypertension or preeclampsia, preterm birth, spontaneous abortion, gestational diabetes mellitus, and fetal development and metabolism.

**Figure 1:**
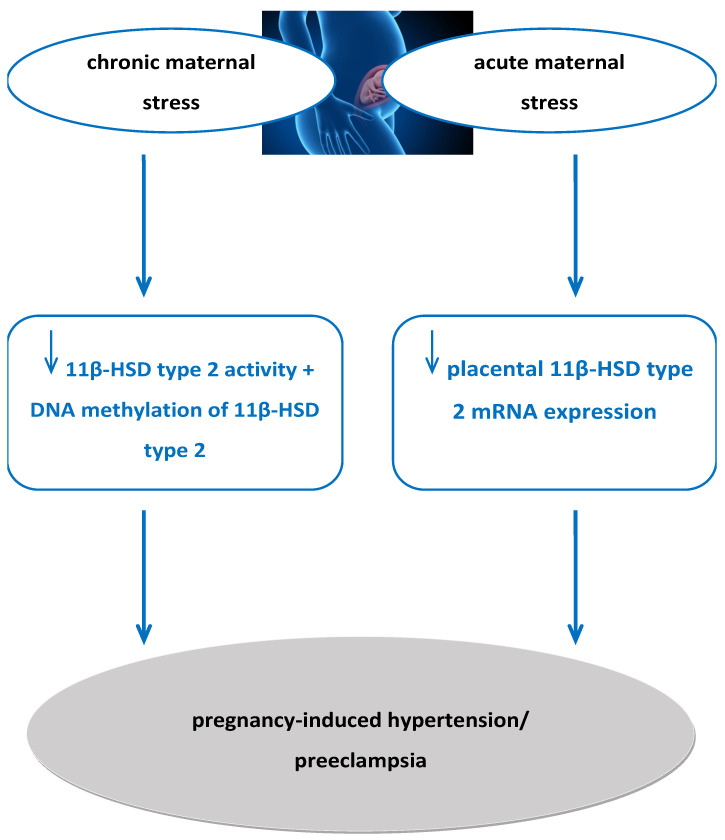
Proposed pathophysiological mechanism of increased maternal stress during pregnancy affecting 11β-HSD type 2 activity and expression, resulting in preeclampsia. 11β-HSD2: 11beta-hydroxysteroid dehydrogenase 2.

Firstly, according to our findings, chronic and acute maternal stress during pregnancy is linked with pregnancy-induced hypertension and preeclampsia, especially when it comes to maternal anxiety during the first trimester of pregnancy [34]. Increased chronic maternal stress during the prenatal period down-regulates 11β-HSD type 2 and alters 11β-HSD type 2 gene expression through DNA methylation [73]. Regarding acute maternal stress during pregnancy, it is shown to be significantly negatively correlated with placental 11β-HSD type 2 mRNA expression [67]. Moreover, 11β-HSD type 2’s expression and activity seem reduced in preeclampsia [83]. Thus, we propose that there is a pathophysiological mechanism through which chronic and acute maternal stress during pregnancy reduces 11β-HSD type 2 function and expression, resulting in pregnancy-induced hypertension and preeclampsia (Figure 1).

**Figure 2:**
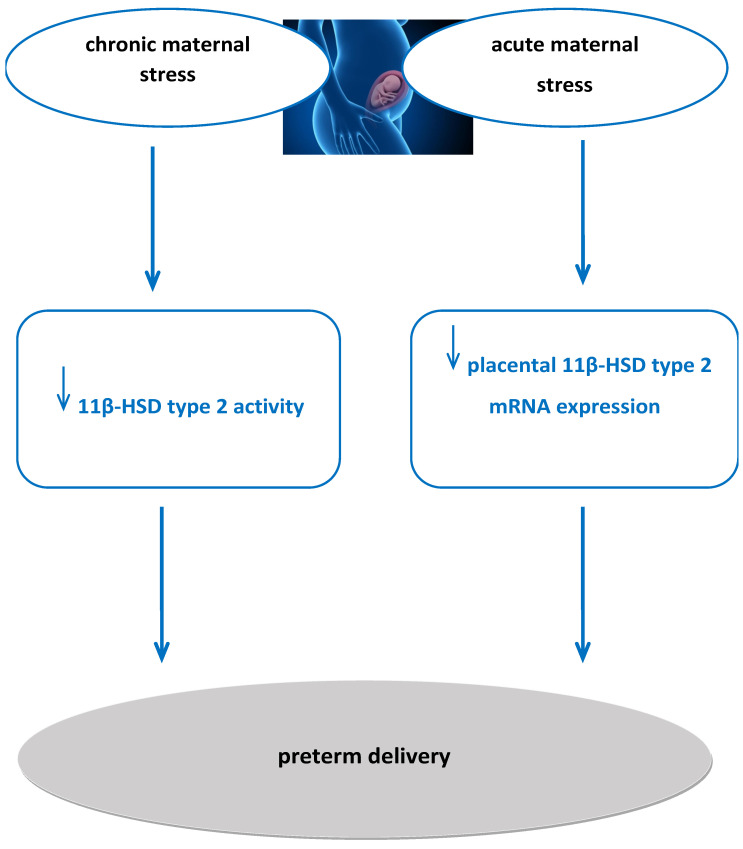
Proposed pathophysiological mechanism of increased maternal stress during pregnancy affecting 11β-HSD type 2 activity and expression, resulting in preterm birth. 11β-HSD2: 11beta-hydroxysteroid dehydrogenase 2.

Furthermore, chronic and acute maternal stress during pregnancy may lead to preterm birth [36]. Increased chronic maternal stress during pregnancy down-regulates 11β-HSD type 2 enzyme [73], while acute maternal stress during pregnancy is significantly negatively correlated with placental 11β-HSD type 2 mRNA expression [67]. As we conclude after our literature search of animal and clinical studies, 11β-HSD type 2 deficiency during pregnancy may result in preterm birth [1]. Therefore, there is enough evidence to conclude that there is a pathophysiological mechanism through which chronic and acute maternal stress during pregnancy reduces 11β-HSD type 2 function and expression, resulting in possible preterm births (Figure 2).

**Figure 3:**
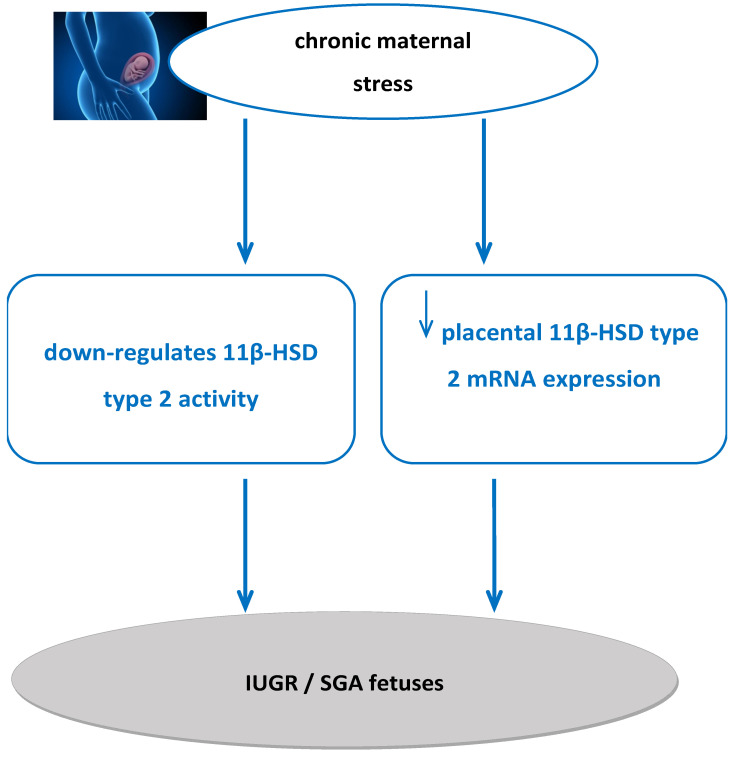
Proposed pathophysiological mechanism of increased maternal stress during pregnancy affecting 11β-HSD type 2 activity and expression, resulting in fetuses with IUGR or SGA. 11β-HSD2: 11beta-hydroxysteroid dehydrogenase 2.

Regarding fetal development, chronic maternal stress during pregnancy may result in abnormal intrauterine growth and IUGR pregnancies [51]. In detail, chronic maternal stress during pregnancy may lead to reduced birth weight, slow growth rate [54,55], and reduced birth length and head circumference [58]. Increased chronic maternal stress during pregnancy down-regulates 11β-HSD type 2 enzyme [73]. Reduced 11β-HSD type 2 activity during pregnancy may result in low birth weight, while reduced placental 11β-HSD type 2 expression also leads to IUGR pregnancies as term approaches [90]. Thus, we propose that there is a pathophysiological mechanism of chronic maternal stress during pregnancy that reduces 11β-HSD type 2 activity and expression, resulting in possible pregnancies with IUGR and births of SGA fetuses (Figure 3).

The potential dysregulation of 11β-HSD isoenzymes and how this results in mother and fetus complications is still under investigation, as it is an emerging area of scientific interest and future research [1]. According to all the data we collected through our literature search, chronic and acute maternal stress during pregnancy is involved in the pathophysiology of several pregnancy complications and fetal developmental and metabolic abnormalities. It is worth mentioning that there is no satisfying published research data regarding 11β-HSD type 1 function and expression, as well as how its dysfunction leads to pregnancy complications and fetal developmental and metabolic abnormalities.

Recent therapeutic strategies suggest that inhibition of 11β-HSD type 1 may provide a potential therapeutic target. Regarding the different effects that trigger delivery, they are attributed to increased concentrations of prostaglandins and also an abundant 11β-HSD type 1 expression. Cortisol produced by increased 11β-HSD type 1 in fetal membranes has several outcomes that lead to delivery. Enhanced activation of 11β-HSD type 1, as in the case of inflammation, may trigger events resulting in preterm birth. Therefore, inhibition of 11β-HSD type 1 in the fetal membranes may provide a potential therapeutic target with the aim of preventing preterm birth [82].

**Figure 4:**
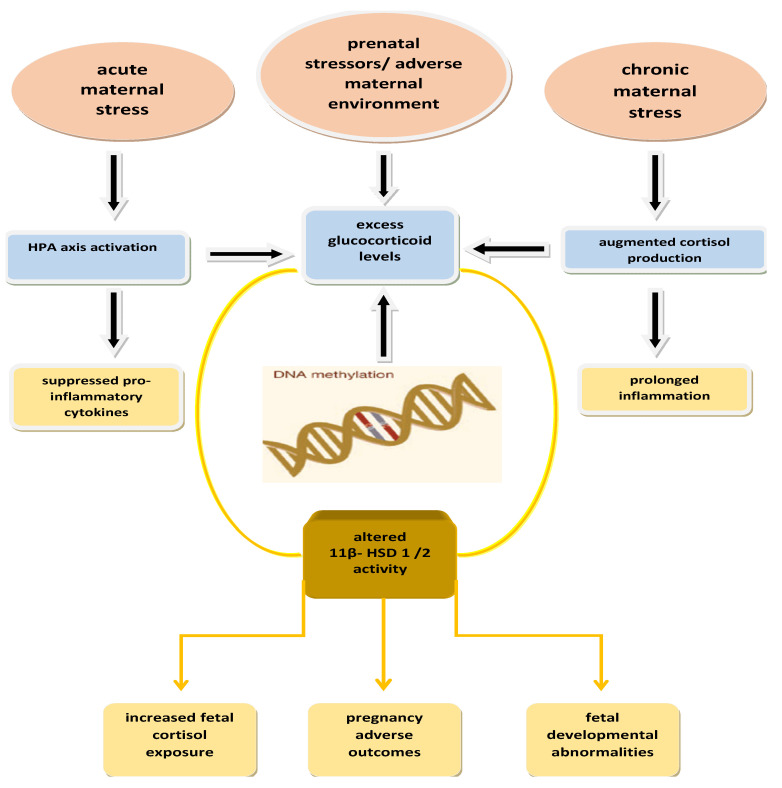
Proposed pathophysiological pathway of increased maternal stress during pregnancy affecting 11β-HSD type 1 and type 2 activity, through HPA axis activation and increased cortisol, leading to increased fetal cortisol exposure, pregnancy complications, and fetal developmental abnormalities. 11β-HSD: 11beta-hydroxysteroid dehydrogenase. HPA axis: hypothalamic-pituitary-adrenal axis.

All things considered, the research data provide evidence that suggests that there is a pathophysiological connection between maternal stress during pregnancy, 11β-HSD dysfunction and adverse pregnancy outcomes, preeclampsia, preterm delivery, and IUGR and SGA newborns (Figure 4). This scientific field provides research potential.

However, more studies are needed to confirm our findings and provide insights into this pathophysiological connection among maternal stress during pregnancy, placental 11β-HSD dysfunction and pregnancy complications, and fetal developmental and metabolic abnormalities. Future research should aim to provide answers about the exact pathophysiological mechanism through which chronic and acute maternal stress during pregnancy directly affects 11β-HSD isoenzyme activity and expression, and especially affecting the 11β-HSD type 1 isoenzyme about which little is known. Moreover, it is necessary for more clinical trials to be carried out regarding the potential therapeutic ability of an inhibitor of 11β-HSD type 1 and to investigate the case that this is safe to be considered during pregnancy.

## 8. Conclusions

In conclusion, convincing data suggest that there is a pathophysiological connection among maternal stress during pregnancy, both chronic and acute, placental 11β-HSD isoenzyme dysfunction, and adverse pregnancy outcomes, particularly preeclampsia, preterm delivery, and IUGR and SGA newborns. Future research should focus on the exact pathophysiological pathway through which chronic and acute maternal stress during pregnancy directly affects placental 11β-HSD isoenzyme activity and expression, particularly the 11β-HSD type 1 isoenzyme, about which little is known. Moreover, upcoming therapeutic targets, such as 11β-HSD 1 inhibitors, should be examined through thorough clinical research. Further research, carefully designed with randomized controlled trials, should illustrate all scientific areas lacking sufficient proof.

## Data Availability

No new data were created or analyzed in this study. Data sharing is not applicable to this article.
